# Implication of Terminal Residues at Protein-Protein and Protein-DNA Interfaces

**DOI:** 10.1371/journal.pone.0162143

**Published:** 2016-09-09

**Authors:** Olivier M. F. Martin, Loïc Etheve, Guillaume Launay, Juliette Martin

**Affiliations:** Univ Lyon, CNRS, UMR 5086 MMSB, 7 passage du Vercors F-69367, Lyon, France; Indian Institute of Science, INDIA

## Abstract

Terminal residues of protein chains are charged and more flexible than other residues since they are constrained only on one side. Do they play a particular role in protein-protein and protein-DNA interfaces? To answer this question, we considered large sets of non-redundant protein-protein and protein-DNA complexes and analyzed the status of terminal residues and their involvement in interfaces. In protein-protein complexes, we found that more than half of terminal residues (62%) are either modified by attachment of a tag peptide (10%) or have missing coordinates in the analyzed structures (52%). Terminal residues are almost exclusively located at the surface of proteins (94%). Contrary to charged residues, they are not over or under-represented in protein-protein interfaces, but strongly prefer the peripheral region of interfaces when present at the interface (83% of terminal residues). The almost exclusive location of terminal residues at the surface of the proteins or in the rim regions of interfaces explains that experimental methods relying on tail hybridization can be successfully applied without disrupting the complexes under study. Concerning conformational rearrangement in protein-protein complexes, despite their expected flexibility, terminal residues adopt similar locations between the free and bound forms of the docking benchmark. In protein-DNA complexes, N-terminal residues are twice more frequent than C-terminal residues at interfaces. Both N-terminal and C-terminal residues are under-represented in interfaces, in contrast to positively charged residues, which are strongly favored. When located in protein-DNA interfaces, terminal residues prefer the periphery. N-terminal and C-terminal residues thus have particular properties with regard to interfaces, which cannot be reduced to their charged nature.

## Introduction

Interactions between proteins or proteins and DNA are a generic process underlying many biological processes. Thanks to the growing number of complexes available in the Protein Data Bank (PDB [1]), generic principles of protein-protein interactions have been discovered [2–8]. Protein-protein binding sites are relatively large and flat; their organization shows as a core of hydrophobic residues surrounded by a rim of polar residues that occlude the solvent. In addition, only a small set of residues contribute most to the free energy of binding. Hetero- and homo- complexes have similar interface properties [7,9], but interfaces from transient complexes tend to be smaller and less hydrophobic than interfaces from permanent complexes [10,11].

In this anatomic description of protein binding sites, nothing has been said concerning the terminal residues of protein chains, which are often hybridized in biochemical techniques aimed at detecting protein-protein interactions. Being the first and last residues of the chain, terminal residues have peculiar properties: they are charged and presumably highly flexible, because they are connected to the rest of the peptidic chain only on one side. These properties have been studied in the context of isolated proteins. It has been shown that terminal residues display some sequence and conformation preferences [12], and reside in sequence regions that are typically enriched in intrinsic disorder [13]. Terminal residues are predominantly located at the surface of proteins, to an extent that cannot be explained only by their charged nature [14]. Lattice model simulations suggested that this location confers the structures kinetic and thermodynamic folding advantages as well as optimal structure compaction [14]. Terminal residues are the first and the last residues of the chain to be synthesized. Krishna and Englander shown that all proteins that fold *via* a two-step mechanism (formation of secondary structures, followed assembly of secondary structures) have their terminal secondary structure elements in contact [15]. This bias toward N-C contacts could relate to the folding mechanism and protein stability [15]. It also explains earlier observations that terminal segments are closer to each other than expected [16,17]. A subsequent study, however, found no sign of directed N to C folding in the final structures [18]. It thus seems that terminal residues have peculiar properties in protein structures.

In protein-DNA complexes, protein tails are thought to play a major role in the recognition [19] and, in some systems, tail composition affects the efficiency of the DNA search process [20]. Protein tails are indeed involved in the specificity and stabilization in several complexes [21,22].

In this paper we specifically study terminal residues in the context of protein-protein and protein-DNA complexes. On a large non-redundant set of dimeric protein-protein interfaces, we quantify their implication in interfaces and its significance compared to random, in different classes of complexes. We also assess whether terminal residues are displaced upon interaction, compared to their location is isolated structures. This is done on a set of protein-protein complexes for which the structures of isolated monomers are available. We then investigate the link between implication at the interface and biochemical hybridization. Finally, we analyze the implication of terminal residues in protein-DNA complexes.

## Results

We took into account the presence and integrity of terminal residues in structures, and computed their frequency at protein-protein interfaces and specific regions of interfaces, in a large dataset of 17,658 binary complexes, non-redundant at the interface level, termed DIMER70, and a filtered dataset of 5,203 proteins, non-redundant at the monomer level, termed MONOMER25. We analyzed their conformational rearrangement upon complexation using the Docking Benchmark [23], the correlation between the presence of both terminals at an interface and the potential link with the experimental techniques for detecting protein-protein interactions. We also analyzed three datasets of protein-DNA structures for the frequency of terminal residues in interfaces and preference for different interface regions.

### More Than Half of Terminal Residues are Disordered or Modified

In order to study the location of terminal residues in protein-protein complexes, we have to take into account the presence of expression tags in the resolved structures, and missing coordinates. Both factors could interfere with our analyses: expression tags add artefactual residues to native protein tails, and missing coordinates prevent any structural analysis.

**Fig 1:**
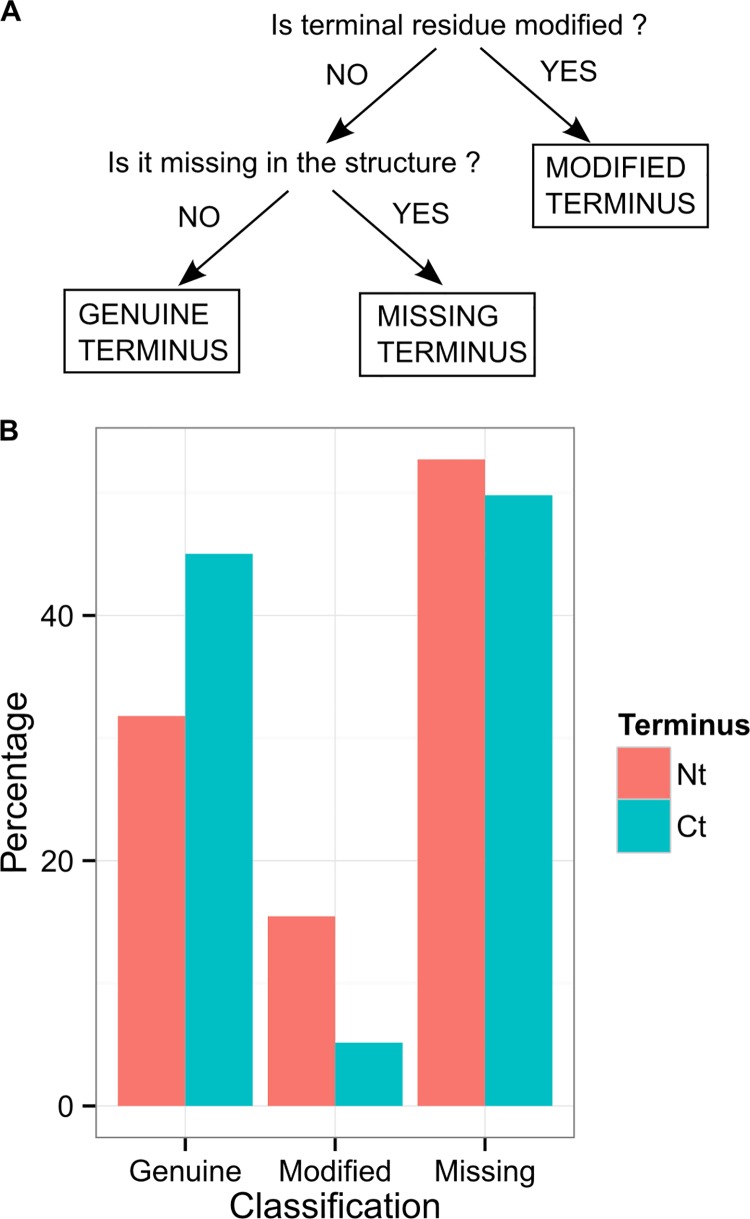
Terminal residues are mostly modified or missing in the structures. A: classification of terminal residues into three categories. Terminal residues are termed *modified* if they are linked to a tag sequence, *missing* if they have no tag, but coordinates are missing from the structure, and *genuine* otherwise. B: division of terminal residues in the three groups, for all the proteins from the DIMER70 dataset (65,284 residues analyzed).

Terminal residues were thus classified into three categories: modified, missing or genuine, as illustrated in Fig 1A. The division of all the N-terminal and C-terminal residues from the DIMER70 data set (65,284 terminal residues successfully analyzed) is shown in Fig 1B. Only 38% of terminal residues are genuine on average, the rest being either modified (10%) or missing (52%). N- and C-terminal residues differ, with less genuine N-terminal residues (32%) than C-terminal (45%). This difference is mainly due to the prevalence of modification of N-terminal residues (15%) compared to C-terminal residues (5%). N-terminal residues are also slightly more often missing (53%) than C-terminal residues (50%). The same trend is observed on the smaller MONOMER25 dataset (9746 terminal residues analyzed from 6235 chains), see Figure A in S1 File.

The classification of all the terminal residues in the DIMER70 dataset is available in S2 File.

In order to study terminal residues in their native structural context, we restrict the rest of our analyses to the genuine class (25,078 terminal residues from the DIMER70 dataset).

### Terminal Residues Are Mostly Located at the Surface of the Proteins

We computed the relative accessible surface area of terminal residues in the context of isolated monomers. Using a 25% of relative accessible area cutoff as in [9], we found that 94% of the genuine terminal residues from the DIMER70 dataset are located at the surface of the proteins, without asymmetry between N-terminal and C-terminal residues. The same proportion is observed for the MONOMER25 dataset. When looking at raw accessibility values N-terminal residues were found marginally more exposed than C-terminal residues (Figure B in S1 File).

### One Quarter of Terminal Residues are Involved in Protein-Protein Interfaces

**Fig 2:**
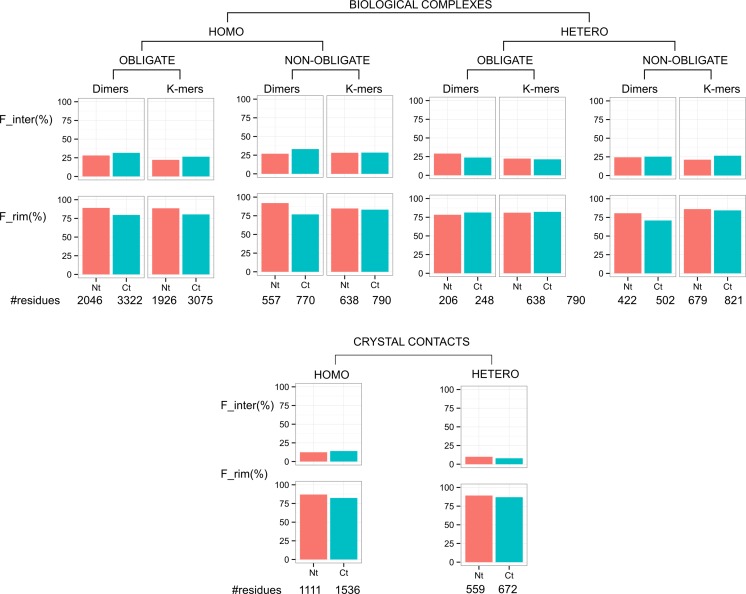
Frequency of terminal residues in interfaces and rim regions, in the DIMER70 data set. #residues: number of terminal residues analyzed, F_inter: fraction of terminal residues involved in interfaces, F_rim: fraction of terminal residues in the rim of the regions, among those involved in interfaces.

We classified residues into different regions (surface, interior, core, rim, support) according to their exposed surface area, in isolated monomers and in complexes, as described in the Materials and Methods section. The amino-acid compositions in different regions are shown in Figure C in S1 File. As previously observed [7], protein surfaces are enriched in polar residues compared to the global composition and interface cores are richer in hydrophobic residues than the rim. On average, 23% of the genuine terminal residues are involved in the protein-protein interfaces in the DIMER70 dataset (21% for N-terminal and 24% for C-terminal). Similar results are obtained for the MONOMER25 data set (see Tables A and B in S1 File). In terms of amino-acid composition, N-terminal residues are depleted in arginine and enriched in methionine residues, while C-terminal residues exhibit less marked preferences (Figure D in S1 File). When taking into account the classification of complexes into different categories (homo- or hetero-complexes, obligate or non-obligate complexes, dimers or K-mers, biological interfaces or crystal contacts, see Materials and Methods), few differences emerge, see Fig 2. The rate of terminal residues in interfaces is higher in biological interfaces than in interfaces due to crystal contacts. Also, the rate is higher in interfaces extracted from dimers *versus* K-mers. This is the direct consequence of a size effect, as explained in the next section.

C-terminal residues are slightly more frequent than N-terminal residues in homo-interfaces, with the exception of non-obligate K-mers. In hetero-interfaces the situation is reversed: the rate is higher for N-terminal residues, again with the exception of non-obligate K-mers. In the next paragraph, we assess the significance of these frequencies, compared to random expectations due to protein size.

### Terminal Residues Are Not Significantly Preferred or Avoided in the Interfaces

In this section, we assess whether the rate of terminal residues in interfaces is different from what is expected by chance. For this, we simulate 1000 data sets with a random model based on hypergeometric laws for each protein, as explained in Materials and Methods. This model takes into account the size of each protein, and the fact that different categories of complexes display different size distributions (see Figure E in S1 File). The simulated data sets allow us to draw empirical distributions for the fraction of residues involved in interfaces, and assess how our observed fractions differ from the random expectation. As shown in Figures F and G in S1 File, the observed fractions of residues in interfaces fall within the simulated ranges for both DIMER70 and MONOMER25 dataset, meaning that there is no over- or under-representation of terminal residues at the protein-protein interfaces.

In other words, the tendency of terminal residues to be involved in interfaces does not deviate from what is expected, given protein size and interface size. Smaller proteins have bigger interfaces (relatively to their sizes), and hence, a higher chance to involve a terminal residue at the interface solely by chance. On the contrary, bigger proteins have smaller (relative) interfaces, and hence, lower probability to have terminal residues involved at the interface.

How does this contrast with the behavior of charged residues? To answer this question, we computed the fraction of charged residues in interfaces (without consideration of their location in the protein chain) and simulated data sets using the same protocol based on hypergeometric laws (see Materials and Methods). Lysine, aspartate and glutamate residues were found under-represented in interfaces, and arginine residues were found over-represented (Figure H in S1 File), in good agreement with previous observations [7,24,25].

In conclusion, terminal residues are not significantly preferred or avoided at protein-protein interfaces, unlike charged residues.

### When involved in interfaces, terminal residues are over-represented in the rim region

**Fig 3:**
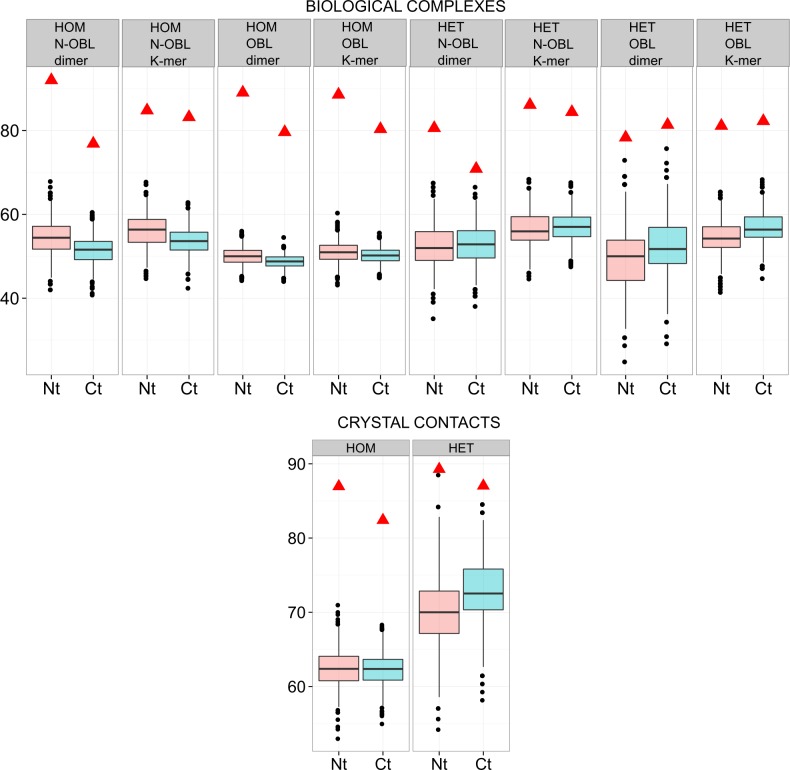
Terminal residues are overrepresented in rim regions in the DIMER70 data set. Each box plot displays the distribution of simulated values of F_rim (fraction of terminal residues in rim regions, among those in interfaces) computed from 1000 random data sets. Box edges correspond to the 25^th^ and 75^th^ percentiles, the notches extend from the 1^st^ to the 99^th^ percentiles, and black points are outliers. Observed fractions are depicted as red triangles. HOM: homo-complexes, HET: hetero-complexes, N-OBL: non-obligate complexes, OBL: obligate complexes. Pink boxes: N-terminal residues, blue boxes: C-terminal residues.

Interfaces were further divided between rim and core regions, based on accessibility values (see Materials and Methods). Most of the terminal residues in interfaces belong to the rim region, i.e., the periphery of the interfaces: 83% of the terminal residues (87% for N-terminal and 81% for C-terminal), see Fig 2. To assess the significance of these numbers we simulated random data sets using hypergeometric laws like before. As can be seen in Fig 3, in this case, the observed proportions are always higher than the simulated ones (empirical p-values<10^−3^), indicating that terminal residues have a preference for rim regions. This preference is confirmed on the MONOMER25 dataset (Figure I in S1 File). It is known that the rim of interfaces is enriched in polar and charged residues [26]. It is thus expected to find an enrichment of terminal residues, since they are charged.

We performed the same analysis on charged residues. The majority of them, when involved in interfaces, are located in the rim, although to a lesser extent than terminal residues: 76% for lysine, 62% for arginine, 66% for aspartate and 69% for glutamate residues. Simulations show that charged residues are, as expected, over-represented in the rim regions (Figure J in S1 File).

**Fig 4:**
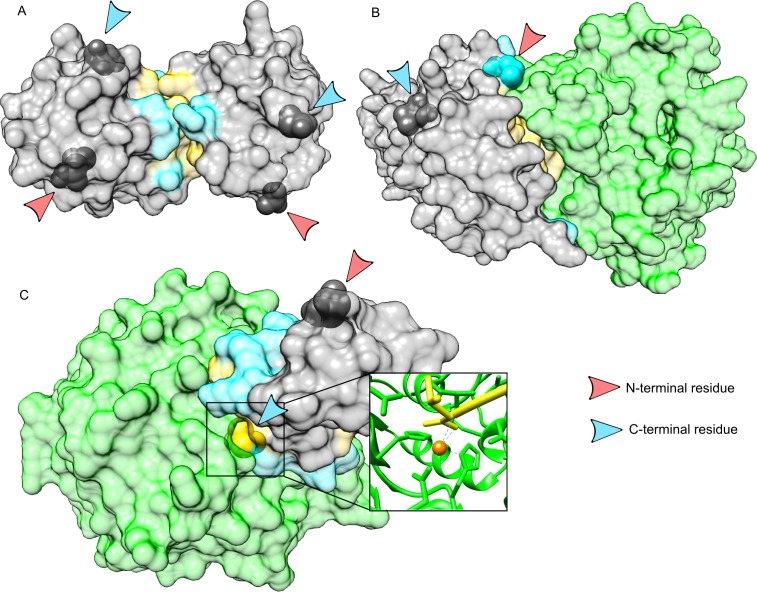
Examples of terminal residue implication at protein-protein interfaces. A: complex between the caspase-recruitment domain of APAF-1 (Apoptotic Protease Activating Factor 1) and the pro-domain of human procaspase-9 (PDB code 3YGS [27]). In this complex, all the terminal residues are genuine, and none of them is involved at the protein-protein interface. B: complex between two subunits of the mRNA capping enzyme of the vaccinia virus (PDB code 2VDW [28]). The genuine N-terminal residue of the small subunit is involved in the rim of the interface. C: complex between a human carboxypeptidase and an inhibitor from leech (PDB code 1DTD [29]). In this complex, the C-terminal residue of the inhibitor is buried at the core of the interface. The close-up view reveals that the terminal carboxylate group of the backbone is interacting with a Zinc ion, which is also coordinated by three side-chains of the enzyme. Interfaces are colored in yellow (core) and cyan (rim). Terminal residues are represented as opaque spheres and highlighted by arrows. Images are generated using UCSF Chimera [30].

Fig 4 presents examples of protein-protein complexes with terminal residues in the three different situations: exposed at the surface of the proteins, but not involved in the interface (Fig 4A), involved in the interface and located in the rim (Fig 4B), and involved in the interface and buried in the interface core (Fig 4C). In the case of terminal residue buried in the core of the interface, we noted several examples similar to the one illustrated in Fig 4C, where the tail of the protein chain interacts with an ion, also coordinated by side chains of the partner. This interaction presumably compensates the energetic penalty induced by burying a charge at the interface.

### Conformational rearrangement of terminal residues upon complexation: insight from the docking benchmark

**Fig 5:**
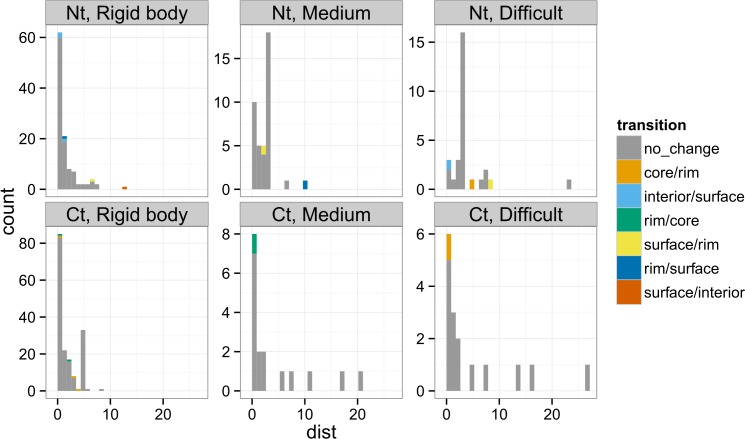
Distance between terminal residues in the Docking Benchmark version 5 after superimposition of bound and unbound forms. Cases are colored according to the interface regions in the unbound/bound conformations. Complexes are classified as ‘rigid body’, ‘medium’ or ‘difficult’ cases in the docking benchmark, based on the structural difference between the bound and the unbound forms.

We analyzed the location of terminal residues in the proteins of the Docking Benchmark 5.0 [31]. This data set contains protein-protein complexes for which the structures of isolated monomers -termed unbound forms- are available in the PDB. For each terminal residue, we compared the location, in terms of interface regions, between the bound and the unbound structures. Among the 381 genuine terminal residues analyzed (180 N-terminal and 201 C-terminal), almost all (363) are classified in the same region (surface/rim/core/support) in bound and unbound structures. We computed the distance between terminal residues after superimposition between bound and unbound forms. In a majority of cases, the distance between terminal residues of bound and unbound structures is very short, see Fig 5. Notably, it is also the case in ‘difficult complexes’, which undergo major structural changes upon complexation. The few distances greater than 10 Å do not affect the classification in regions (surface residues that remain at surface after complexation). Reallocation of terminal residues to a different region upon complexation thus appears to be a rare event.

### Co-occurrence of Terminal Residues in Interfaces

In this section, we ask the following question: do terminal residues co-occur at protein-protein interfaces? We counted the co-occurrence of N-terminal and C-terminal residues at protein-protein interfaces, in the DIMER70 data set, and computed a Chi-squared test to assess the independence, see Table 1.

**Table 1:** Co-occurrence of terminal residues in interfaces, in the DIMER70 data set.

Co-occurrence	COMPLEX type	p-value	#obs/#expected
Intra-chain		**5.5e-14**	**1.34**
Inter-chain	HOMO	**1.8e-13**	**1.38**
Inter-chain	HETERO	0.37	0.86

The p-value is the one of the Chi-squared test on the contingency table counting the frequency of each terminal residue at the interface (intra-chain: terminal residues from the same monomer, inter-chain: terminal residues from different monomers). #obs/#expected is the ratio of observed *versus* expected frequencies of co-presence of terminal residues in interfaces in biological complexes.

We found that the co-occurrence of terminal residues from a same chain is favored (p-value = 5.5e-14), although this preference is modest (ratio of observed *versus* expected = 1.34). This confirms a previous observation that tails tend to be close to each other [17]. When found a significant co-occurence of terminal residues from different chains in homo-dimeric interfaces: terminal residues co-occur in interfaces more often than expected. This is linked to the previous observations: since terminal residues from the same chain tend to co-occur at interfaces (due to their proximity), by symmetry it will also favor the co-occurrence across different chains. By contrast, there is no significant co-occurrence in the case of terminal residues from partner chains across the interface for heterodimers. Data were insufficient to derive meaningful statistics from the MONOMER25 dataset.

### Does Terminal Residue Modification Impair Interaction Detection?

We retrieved, for each complex in the DIMER70 data set, the experimental methods used to detect the interaction, from the IntAct database. We separated the interactions between those detected using methods that require hybridization of tag peptides (such as TAP-tag purification) or fusion domains (such as two-hybrid), and those detected using other methods. We thus had to map from InterEvol chains, through PDB chains, to Uniprot chains. We then searched IntAct for the experimental methods used to detect the interactions. In order to get meaningful results, we restricted our analysis to biological interfaces and genuine residues. Out of the 7079 complexes considered, IntAct provides information for 849 complexes (12%), with a better coverage for hetero-complexes (615 out of 1823, i.e. 33%) than for homo-complexes (234 out of 5256; i.e. 4%).

Hybridization-based techniques require the modification of terminal residues by attachment of a peptide or a protein domain. We hypothesized that this modification could perturb the formation of a complex when terminal residues are in interfaces. Such binary interactions could be more difficult to detect by hybridization-based techniques. In this case, we would expect fewer interactions detected by hybridization-based techniques when terminal residues are in interfaces. Contrary to our expectation, we did not find any such bias (Chi-squared test on the contingency table, p-value = 0.3). Dividing the data set between homo-complexes and hetero-complexes yielded the same result. Let us note that all complexes with a terminal residue in the interface are treated in the same way, since the side on which the hybridization takes place is not reported in IntAct. This factor could weaken the signal, if any. However, we observe here no signal at all. We postulate that this is due to the particular location of terminal residues in interfaces. As we have shown in this study, terminal residues, when involved in interfaces, are preferentially located in the rim. This preferred location presumably could allow the hybridization of tag peptides or fusion domains without disrupting the complex.

### Terminal Residues at Protein-DNA Interfaces

We analyzed the location of terminal residues in two datasets prepared by us: 156 transcription factors complexed with DNA and 265 DNA-binding proteins with other functions. We found that 16% of the genuine N-terminal and 4% of the genuine C-terminal residues were involved in protein-DNA interfaces in transcription factors, *versus* 6% of genuine N-terminal and 3% of the genuine C-terminal in the other DNA-binding proteins. Thus, N-terminal residues seem more frequent in protein-DNA interfaces than C-terminal ones, both in transcription factors and DNA-binding proteins with other functions. In these data sets, no filtering was carried out to reduce the redundancy between protein sequences.

**Fig 6:**
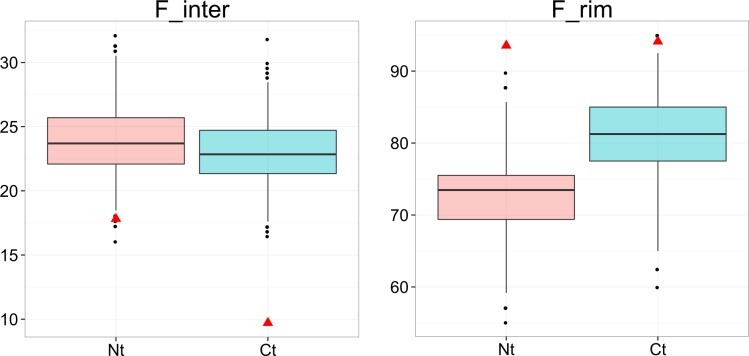
Terminal residues are underrepresented in protein-DNA interfaces, but when involved in interfaces, they are over-represented in rim regions. Data are collected on a list of 303 protein-DNA complexes, non-redundant at the 70% level. F_inter: fraction of terminal residues in interfaces. F_rim: fraction of terminal residues in rim regions, among those in interfaces. Each box plot displays the distribution of simulated values computed from 1000 random data sets. Box edges correspond to the 25^th^ and 75^th^ percentiles, the notches extend from the 1^st^ to the 99^th^ percentiles, and black points are outliers. Observed fractions are depicted as red triangles.

In order to get statistics on non-redundant data, we analyzed the location of terminal residues in a dataset prepared by [32] composed of 303 protein-DNA complexes, irrespective of their function, non-redundant at the 70% level (the sparsity of the PDB does not allow to reduce further the redundancy). In these structures, 174 N-terminal and 175 C-terminal residues are genuine. Overall, 18% of the genuine N-terminal residues and 9% of the genuine C-terminal residues are involved in protein-DNA interfaces. This confirms the preference of N-terminal over C-terminal residues seen in the redundant data sets of transcription factors and other DNA-binding proteins. This preference agrees with the stabilizing electrostatic interaction between the negatively charged DNA phosphate groups and the positively charged amine groups of the N-terminal residues. When comparing observed to expected frequencies, we found that both terminal residues are clearly under-represented at protein-DNA interfaces, see Fig 6. This is in sharp contrast with what happens in protein-protein interface, and contrary to what is expected for N-terminal residues whose positive charge should favor interaction with DNA. To confirm this observation, we filtered out partial structures where the N-terminal residue is not the one of the native protein. We obtained the same result, see Figure K in S1 File. When considering only the small fraction of terminal residues in interfaces, terminal residues are overrepresented in rim regions: the observed frequencies are greater than simulated distributions.

**Fig 7:**
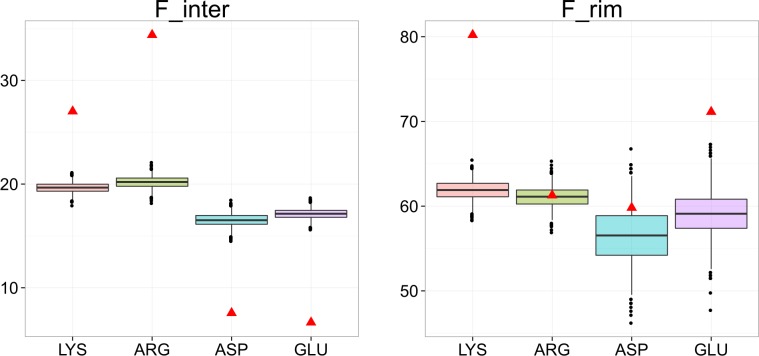
Frequency of charged residues in protein-DNA interfaces. Data are collected on a list of 303 protein-DNA complexes, non-redundant at the 70% level. F_inter: fraction of residues of a given type in interfaces. F_rim: fraction of residues of a given type in rim regions, among those in interfaces. Each box plot displays the distribution of simulated values computed from 1000 random data sets. Box edges correspond to the 25^th^ and 75^th^ percentiles, the notches extend from the 1^st^ to the 99^th^ percentiles, and black points are outliers. Observed fractions are depicted as red triangles. Pink boxes: lysine residues, green boxes: arginine residues, blue boxes: aspartate residues, purple boxes: glutamate residues.

We compared the trend seen for terminal residues to the trend of charged residues, see Fig 7. We found that terminal residues and charged residues do not follow the same trend: positively charged residues lysine and arginine are clearly preferred in interfaces, contrary to N-terminal residues. This preference of lysine and arginines for the protein-DNA interface has been described in an earlier study [33]. Here, we further show that lysine and glutamate residues clearly prefer the rim regions, but not arginine and aspartate residues. Arginine residues are known to play a particular role in protein-DNA interactions, due to their capacity to form bidentate interactions with DNA [34]; it is thus logical to find them also in the core of protein-DNA interfaces.

**Fig 8:**
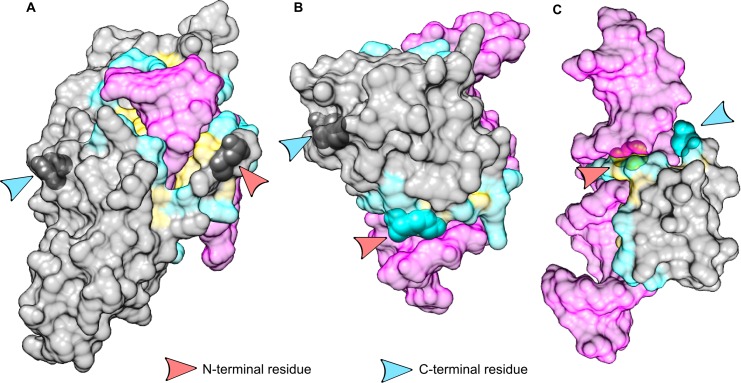
Examples of terminal residue implication at DNA-protein interfaces. A: complex between the restriction endonuclease HinP1 and DNA (PDB code 2FKC [35]). In this complex, protein terminal residues are genuine and none of them is involved in the DNA-protein interface. Orange spheres are calcium ions. B: complex between transcription factor PU.1 and DNA (PDB code 1PUE [36]). The N-terminal residue of the transcription factor (depicted in cartoon representation) is involved at the rim of the interface with DNA (pink surface). C: complex between the DNA binding domain of the *C*. *elegans* Tc3 transposase and DNA (PDB code 1TC3 [37]). The N-terminal residue of the protein is buried at the core of the interface and the C-terminal residue is involved at the rim of the interface. Protein interface is colored in yellow (core region) and cyan (rim regions). Terminal residues are represented as opaque spheres and highlighted by arrows. Images are generated using UCSF Chimera [30].

Fig 8 displays several examples of terminal residue involvement in protein-DNA interfaces.

## Conclusion

In conclusion, our analysis shows that more than half of the terminal residues in protein-protein complex structures from the PDB are either modified or missing. It is thus mandatory to take it into account to perform a fair analysis of terminal residues. Terminal residues do not behave strictly as charged residues. In protein-protein interfaces, they are globally not preferred or avoided in interfaces, while charged residues are clearly under-represented (for lysine, aspartate and glutamate) or over-represented (aspartate). When involved in interfaces, terminal residues prefer the rim region, but their overwhelming presence in the rim cannot be explained only by their charged nature. The particular location of terminal residues in the rim explains why high-throughput techniques that rely on tail hybridization can be successfully applied without abrogating the interaction under study.

The preference of terminal residues for surface has been explained by an advantage in folding and stability [14], suggesting a positive selection of structures with surface terminal residues during evolution. But why are they so strongly favored in the rim? We propose the following hypothesis: steered dynamic simulations suggested that interface residues tend to be less mobile than the rest of the surface residues [38]. If high residue mobility is unfavorable at protein-protein interface, then terminal residues, which are highly flexible in nature, should be avoided at the interfaces, which might be extremely limiting in evolutionary terms. Their segregation in the rim region might be a good trade-off to accommodate their presence at interfaces, without excluding them from the interface.

In protein-DNA complexes, we also observed a contrast between terminal residues and charged residues: terminal residues are all under-represented in protein-DNA interfaces, while charged residues are clearly discriminated according to their charge (over-representation of lysine and arginine and under-representation of aspartate and glutamate). When involved in interfaces, terminal residues also clearly prefer the rim region. Terminal residues thus have specific properties with regard to protein-protein and protein-DNA interfaces that cannot be reduced only to their charge.

## Materials and Methods

### Data set

#### Protein-protein interfaces

An initial list of 17,658 binary interfaces was retrieved from the InterEvol resource [39] (http://biodev.cea.fr/interevol/downloads.html). This list is non-redundant at the dimer level, at the 70% threshold. We termed this list DIMER70. In this list, interfaces are already annotated in terms of:

- heterodimer or homodimer complexes,
- obligate or non-obligate complexes (predicted by NOXclass [40]),
- biological interfaces or crystal contacts (predicted by NOXclass).

We further made the distinction between interfaces extracted from complexes with two chains (dimers) and those extracted from higher order complexes (K-mers), from information available in the PDB.

We filtered the DIMER70 list in order to reduce the redundancy between monomers to 25%, using PISCES (http://dunbrack.fccc.edu/PISCES.php) [41] with the following input parameters: sequence percentage identity <25%, resolution <3Å, R-factor <0.3, sequence length between 40 and 10,000, include non-X-ray structures, exclude CA-only structures, Cull PDB by chain. The filtered list contains 5203 monomers and is termed MONOMER25.

#### Docking benchmark

The docking benchmark version 5.0 was used (https://zlab.umassmed.edu/benchmark/) [31]. Complexes with more than two proteins and proteins with sequence variations between bound and free forms affecting the terminal residues were removed. Terminal residues were classified in the regions defined by Levy [9] in the complexes, and in “unbound complexes” formed by unbound forms superimposed onto complexes. The distance between Cα of terminal residues between bound and free forms was computed to quantify the conformational rearrangement.

#### Protein-DNA complexes

Protein-DNA complex structures solved by X-ray crystallography at a resolution better than 2.60 Å and a maximum R free value of 0.4. were obtained from the PDB. The complexes were defined as any structures containing at least one protein chain, and a double-stranded DNA longer than six base pairs. All entries containing significantly modified DNA were discarded. The list was then split between 156 transcription factors and 265 enzymes.

Another list of protein-DNA complexes with various functions were retrieved from [32], and filtered using PISCES using the following parameters: sequence percentage identity <70%, resolution <3Å, R-factor <0.3, sequence length between 40 and 10,000, include non-X-ray structures, exclude Cα-only structures, cull PDB by chain. This filtered list contained 304 protein chains, from 278 PDB entries.

#### IntAct

We retrieved interaction data from the IntAct resource [42]. The xml files contained in the pmidMIF25.zip archive were parsed to retrieve interactions related to the PDB structures of the DIMER70 dataset. The following experimental methods were considered implying tail modification: adenylate cyclase complementation, affinity technology, anti-bait coimmunoprecipitation, anti-tag coimmunoprecipitation, bimolecular fluorescence complementation, dihydrofolate reductase reconstruction, pull down, two-hybrid, two-hybrid array, two-hybrid fragment pooling approach, two-hybrid pooling approach, ubiquitin reconstruction. The correspondence between PDB chains and Uniprot IDs was performed using the PDB/Uniprot Mapping resource (http://bioinf.org.uk/pdbsws/) [43].

### Structure analysis

#### MMCIF files

Missing coordinates and Tag residues were identified thanks to the mmCIF files from the PDB [1].

#### Naccess

The software NACCESS [44] was used to compute solvent accessible surface area (ASA) using default parameters. Surface residues are defined as those with a relative accessible surface area (RASA) greater than 25% as in [9].

#### Interface definition

Following the definition of Levy [9], residues were classified into the following regions, according to the values of RASA in the monomers (RASAm) and in the complexes (RASAc):

- Non-interface residues (RASAm = RASAc):
  ○ Surface residues (RASAm>25%)
  ○ Interior residues (RASAm <25%)
- Interface residues (RASAm> RASAc):
  ○ Rim (RASAc>25%)
  ○ Core (RASAc <25% and RASAm>25%)
  ○ Support (RASAc <25% and RASAm <25%)

This cutoff of 25% allows an optimal separation between surface and interior amino-acids, in terms of composition [9].

### Statistical analysis

All statistical analyses are made using the R statistical environment [45].

#### Random model

We considered a random model based on the hypergeometric law to assess the significance of the involvement of terminal residues at the interfaces and rim regions.

A hypergeometric law H (k, K, N) describes the number of successes in a trial experiment, where one draws, without replacement, k individuals from a population of size N containing K successes. We used this law to simulate the presence of terminal residues at the interfaces and the rim regions of interfaces. When simulating the presence of a terminal residue at the protein-protein interface, we simulated hypergeometric variables for each protein with parameters set to k = number of interface residues (only rim and core residues), K = 1 (one N-terminal or C-terminal residue), and N = number of surface residues when the monomer is isolated.

To simulate the appearance of a terminal residue at the rim of an interface, we used parameters k = 1, K = number of residues in the rim regions, and N = number of residues at the interface (only rim and core residues).

The reason for excluding support and interior residues is that we have found that almost all terminal residues are exposed to the solvent when monomers are considered alone.

Thus, it was possible, for each protein, to simulate the appearance of terminal residues in interfaces, and, for those proteins with terminal residues in interfaces, their appearance in the rim region. We simulated separate distributions for N-terminal and C-terminal residues, because their statistics are based on different protein subsets (some proteins have only one genuine terminal residue).

The same protocol was used to study the preference of charged residues, without consideration of their location in the chains (i.e. terminal or not). To be consistent with the terminal residue analysis, buried residues (support and interior regions) were excluded from the analysis. For example, when simulating the frequency of arginine residues in interfaces, parameters were set to k = number of interface residues (core and rim), K = number of exposed arginine residues (in surface, core and rim) and N = number of exposed residues, and for simulating the frequency in rim regions, k = number of rim residues, K = number of interface arginines (core and rim) and N = number of interface residues (core and rim).

The use of such random models to simulate data sets results in empirical p-values: when simulating 1000 values, an observed value outside of the simulated range corresponds to an empirical p-value lower than 10^−4^.

## Supporting Information

S1 File Figures A to K and Tables A and B.
(PDF)

S2 File classification of terminal residues from the DIMER70 dataset. (TXT)

## References

[1] Berman H, Henrick K, Nakamura H. Announcing the worldwide Protein Data Bank. Nat Struct Mol Biol. 2003;10: 980–980. 10.1038/nsb1203-980 14634627

[2] Chothia C, Janin J. Principles of protein–protein recognition. Nature. 1975;256: 705–708. 10.1038/256705a0 1153006

[3] Jones S, Thornton JM. Principles of protein-protein interactions. Proc Natl Acad Sci U S A. 1996;93: 13–20. 8552589 10.1073/pnas.93.1.13 PMC40170

[4] Chakrabarti P, Janin J. Dissecting protein–protein recognition sites. Proteins Struct Funct Bioinforma. 2002;47: 334–343. 10.1002/prot.10085 11948787

[5] Yan C, Wu F, Jernigan RL, Dobbs D, Honavar V. Characterization of Protein–Protein Interfaces. Protein J. 2008;27: 59–70. 10.1007/s10930-007-9108-x 17851740 PMC2566606

[6] Bahadur RP, Zacharias M. The interface of protein-protein complexes: analysis of contacts and prediction of interactions. Cell Mol Life Sci CMLS. 2008;65: 1059–1072. 10.1007/s00018-007-7451-x 18080088 PMC11131830

[7] Talavera D, Robertson DL, Lovell SC. Characterization of Protein-Protein Interaction Interfaces from a Single Species. PLoS ONE. 2011;6: e21053 10.1371/journal.pone.0021053 21738603 PMC3124478

[8] Mezei M. Statistical Properties of Protein-Protein Interfaces. Algorithms. 2015;8: 92–99. 10.3390/a8020092

[9] Levy ED. A Simple Definition of Structural Regions in Proteins and Its Use in Analyzing Interface Evolution. J Mol Biol. 2010;403: 660–670. 10.1016/j.jmb.2010.09.028 20868694

[10] Block P, Paern J, Hüllermeier E, Sanschagrin P, Sotriffer CA, Klebe G. Physicochemical descriptors to discriminate protein-protein interactions in permanent and transient complexes selected by means of machine learning algorithms. Proteins. 2006;65: 607–622. 10.1002/prot.21104 16955490

[11] Perkins JR, Diboun I, Dessailly BH, Lees JG, Orengo C. Transient Protein-Protein Interactions: Structural, Functional, and Network Properties. Structure. 2010;18: 1233–1243. 10.1016/j.str.2010.08.007 20947012

[12] Pal D, Chakrabarti P. Terminal residues in protein chains: Residue preference, conformation, and interaction. Biopolymers. 2000;53: 467–475. 10.1002/(SICI)1097-0282(200005)53:6<467::AID-BIP3>3.0.CO;2-9 10775062

[13] Uversky VN. The most important thing is the tail: Multitudinous functionalities of intrinsically disordered protein termini. FEBS Lett. 2013;587: 1891–1901. 10.1016/j.febslet.2013.04.042 23665034

[14] Jacob E, Unger R. A tale of two tails: why are terminal residues of proteins exposed? Bioinformatics. 2007;23: e225–e230. 10.1093/bioinformatics/btl318 17237096

[15] Krishna MMG, Englander SW. The N-terminal to C-terminal motif in protein folding and function. Proc Natl Acad Sci U S A. 2005;102: 1053–1058. 10.1073/pnas.0409114102 15657118 PMC545867

[16] Thornton JM, Sibanda BL. Amino and carboxy-terminal regions in globular proteins. J Mol Biol. 1983;167: 443–460. 10.1016/S0022-2836(83)80344-1 6864804

[17] Christopher JA, Baldwin TO. Implications of N and C-Terminal Proximity for Protein Folding. J Mol Biol. 1996;257: 175–187. 10.1006/jmbi.1996.0154 8632453

[18] Laio A, Micheletti C. Are structural biases at protein termini a signature of vectorial folding? Proteins Struct Funct Bioinforma. 2006;62: 17–23. 10.1002/prot.20712 16281293

[19] Agnes Tóth-Petróczy IS. Disordered tails of homeodomains facilitate DNA recognition by providing a trade-off between folding and specific binding. J Am Chem Soc. 2009;131: 15084–5. 10.1021/ja9052784 19919153

[20] Chatr-Aryamontri A, Breitkreutz B-J, Oughtred R, Boucher L, Heinicke S, Chen D, et al The BioGRID interaction database: 2015 update. Nucleic Acids Res. 2015;43: D470–D478. 10.1093/nar/gku1204 25428363 PMC4383984

[21] Joshi R, Passner JM, Rohs R, Jain R, Sosinsky A, Crickmore MA, et al Functional specificity of a Hox protein mediated by the recognition of minor groove structure. Cell. 2007;131: 530–543. 10.1016/j.cell.2007.09.024 17981120 PMC2709780

[22] Santelli E, Richmond TJ. Crystal structure of MEF2A core bound to DNA at 1.5 Å resolution1. J Mol Biol. 2000;297: 437–449. 10.1006/jmbi.2000.3568 10715212

[23] Hwang H, Vreven T, Janin J, Weng Z. Protein-protein docking benchmark version 4.0. Proteins Struct Funct Bioinforma. 2010;78: 3111–3114. 10.1002/prot.22830 PMC2958056 20806234

[24] Jones S, Thornton JM. Analysis of protein-protein interaction sites using surface patches. J Mol Biol. 1997;272: 121–132. 10.1006/jmbi.1997.1234 9299342

[25] Crowley PB, Golovin A. Cation–π interactions in protein–protein interfaces. Proteins Struct Funct Bioinforma. 2005;59: 231–239. 10.1002/prot.20417 15726638

[26] Martin J, Lavery R. Arbitrary protein-protein docking targets biologically relevant interfaces. BMC Biophys. 2012;5: 7 10.1186/2046-1682-5-7 22559010 PMC3441232

[27] Qin H, Srinivasula SM, Wu G, Fernandes-Alnemri T, Alnemri ES, Shi Y. Structural basis of procaspase-9 recruitment by the apoptotic protease-activating factor 1. Nature. 1999;399: 549–557. 10.1038/21124 10376594

[28] De la Peña M, Kyrieleis OJP, Cusack S. Structural insights into the mechanism and evolution of the vaccinia virus mRNA cap N7 methyl-transferase. EMBO J. 2007;26: 4913–4925. 10.1038/sj.emboj.7601912 17989694 PMC2099473

[29] Reverter D, Fernández-Catalán C, Baumgartner R, Pfänder R, Huber R, Bode W, et al Structure of a novel leech carboxypeptidase inhibitor determined free in solution and in complex with human carboxypeptidase A2. Nat Struct Biol. 2000;7: 322–328. 10.1038/74092 10742178

[30] Pettersen EF, Goddard TD, Huang CC, Couch GS, Greenblatt DM, Meng EC, et al UCSF Chimera—a visualization system for exploratory research and analysis. J Comput Chem. 2004;25: 1605–1612. 10.1002/jcc.20084 15264254

[31] Vreven T, Moal IH, Vangone A, Pierce BG, Kastritis PL, Torchala M, et al Updates to the Integrated Protein-Protein Interaction Benchmarks: Docking Benchmark Version 5 and Affinity Benchmark Version 2. J Mol Biol. 2015;427: 3031–3041. 10.1016/j.jmb.2015.07.016 26231283 PMC4677049

[32] Schneider B, Černý J, Svozil D, Čech P, Gelly J-C, Brevern AG de. Bioinformatic analysis of the protein/DNA interface. Nucleic Acids Res. 2013; gkt1273. 10.1093/nar/gkt1273 PMC3950675 24335080

[33] Lejeune D, Delsaux N, Charloteaux B, Thomas A, Brasseur R. Protein–nucleic acid recognition: Statistical analysis of atomic interactions and influence of DNA structure. Proteins Struct Funct Bioinforma. 2005;61: 258–271. 10.1002/prot.20607 16121397

[34] Coulocheri SA, Pigis DG, Papavassiliou KA, Papavassiliou AG. Hydrogen bonds in protein–DNA complexes: Where geometry meets plasticity. Biochimie. 2007;89: 1291–1303. 10.1016/j.biochi.2007.07.020 17825469

[35] Horton JR, Zhang X, Maunus R, Yang Z, Wilson GG, Roberts RJ, et al DNA nicking by HinP1I endonuclease: bending, base flipping and minor groove expansion. Nucleic Acids Res. 2006;34: 939–948. 10.1093/nar/gkj484 16473850 PMC1363774

[36] Kodandapani R, Pio F, Ni CZ, Piccialli G, Klemsz M, McKercher S, et al A new pattern for helix-turn-helix recognition revealed by the PU.1 ETS-domain-DNA complex. Nature. 1996;380: 456–460. 10.1038/380456a0 8602247

[37] van Pouderoyen G, Ketting RF, Perrakis A, Plasterk RH, Sixma TK. Crystal structure of the specific DNA-binding domain of Tc3 transposase of C.elegans in complex with transposon DNA. EMBO J. 1997;16: 6044–6054. 10.1093/emboj/16.19.6044 9312061 PMC1170234

[38] Kuttner YY, Engel S. Protein Hot Spots: The Islands of Stability. J Mol Biol. 2012;415: 419–428. 10.1016/j.jmb.2011.11.009 22100447

[39] Faure G, Andreani J, Guerois R. InterEvol database: exploring the structure and evolution of protein complex interfaces. Nucleic Acids Res. 2011;40: D847–D856. 10.1093/nar/gkr845 22053089 PMC3245184

[40] Zhu H, Domingues FS, Sommer I, Lengauer T. NOXclass: prediction of protein-protein interaction types. BMC Bioinformatics. 2006;7: 27 10.1186/1471-2105-7-27 16423290 PMC1386716

[41] Wang G, Dunbrack RL Jr. PISCES: a protein sequence culling server. Bioinforma Oxf Engl. 2003;19: 1589–1591. 10.1093/bioinformatics/btg224 12912846

[42] Orchard S, Ammari M, Aranda B, Breuza L, Briganti L, Broackes-Carter F, et al The MIntAct project—IntAct as a common curation platform for 11 molecular interaction databases. Nucleic Acids Res. 2014;42: D358–D363. 10.1093/nar/gkt1115 24234451 PMC3965093

[43] Martin ACR. Mapping PDB chains to UniProtKB entries. BIOINFORMATICS. 2005;21: 4297–4301. 16188924 10.1093/bioinformatics/bti694

[44] Hubbard SJ, Thornton JM. “NACCESS”, computer programm Department of Biochemistry and Molecular Biology, University College London;

[45] R Development Core Team. R: A Language and Environment for Statistical Computing. Vienna, Austria: R Foundation for Statistical Computing; 2011.

