# Supplementary material for: Implication of Terminal Residues at Protein-Protein and Protein-DNA Interfaces
Source: PLoS One. 2016 Sep 9;11(9):e0162143. doi: 10.1371/journal.pone.0162143 (PMC5017611; doi:10.1371/journal.pone.0162143)
Supplement: S1 File — Figures A to K and Tables A and B. (PDF) [file pone.0162143.s001.pdf]

Supplementary information for "Implication of terminal residues at protein-protein interfaces"

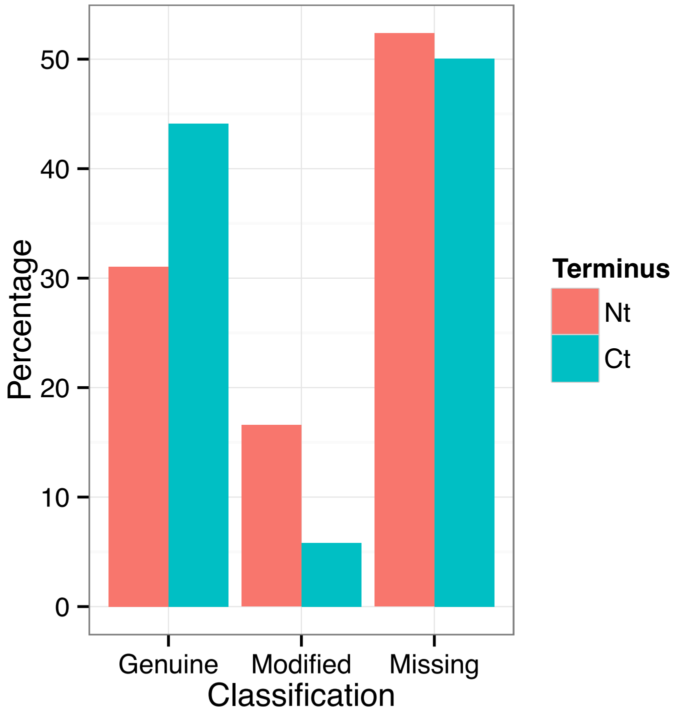

Figure A: Classification of terminal residues into Genuine/Modified/Missing in the MONOMER25 dataset (9746 residues analyzed).

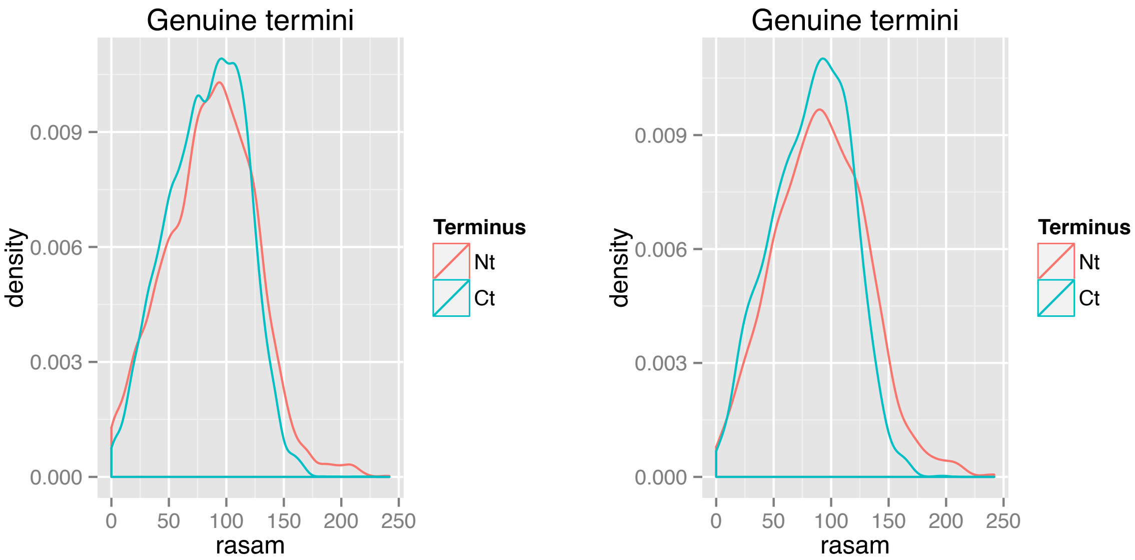

Figure B: Accessibility value distributions for genuine N-ter and C-terminal residues in the DIMER70 dataset (left) and in the MONOMER25 dataset (right). Rasam: relative accessible surface area in the monomeric state.

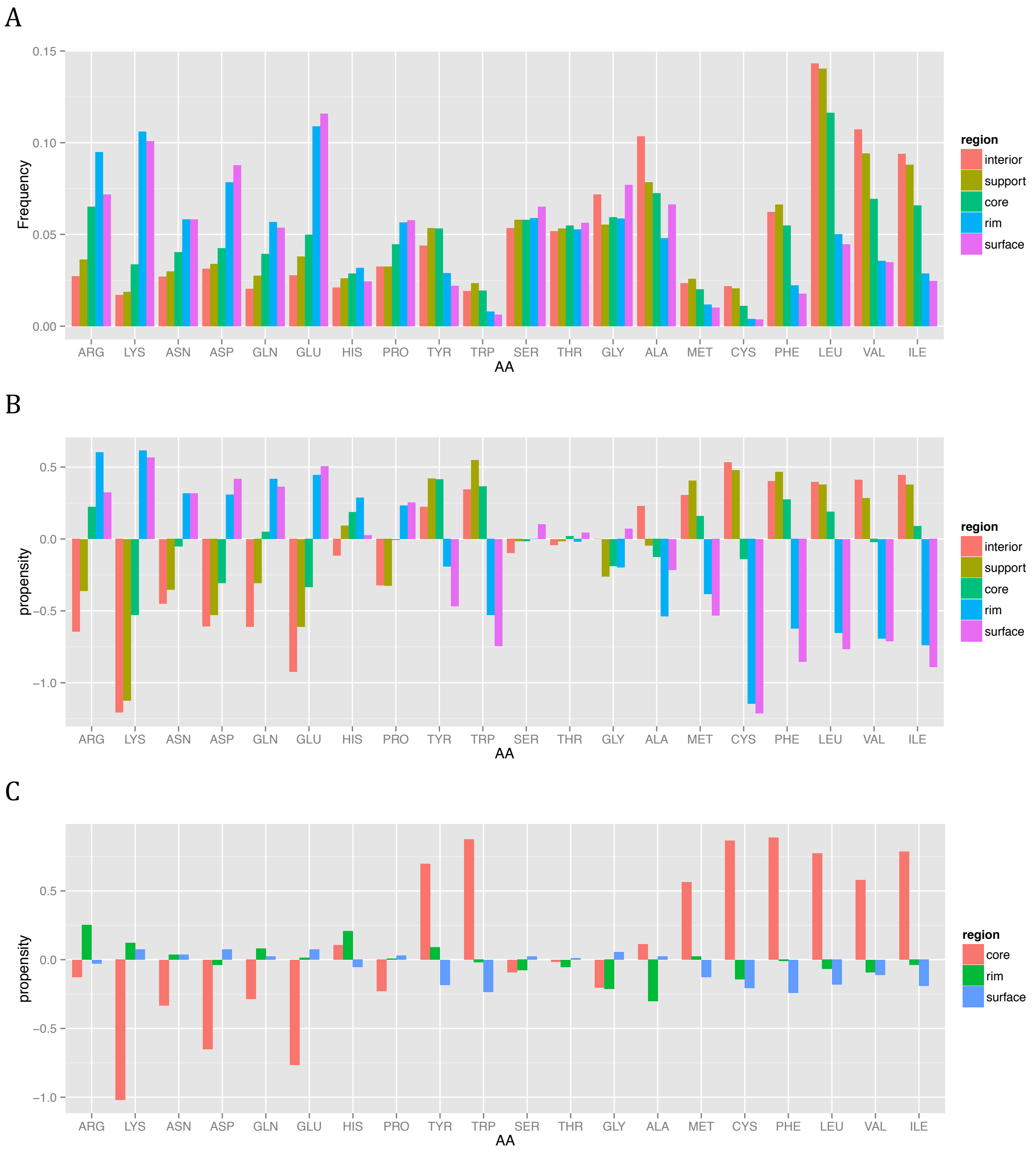

Figure C. Amino-acid composition in the different regions of proteins in the MONOMER25 dataset. A: raw percentages, B: propensities (expressed as log(F/Fref)) compared to the global amino-acid composition, C: propensities in rim core and surface regions compared to the amino-acid composition of exposed residues.

A

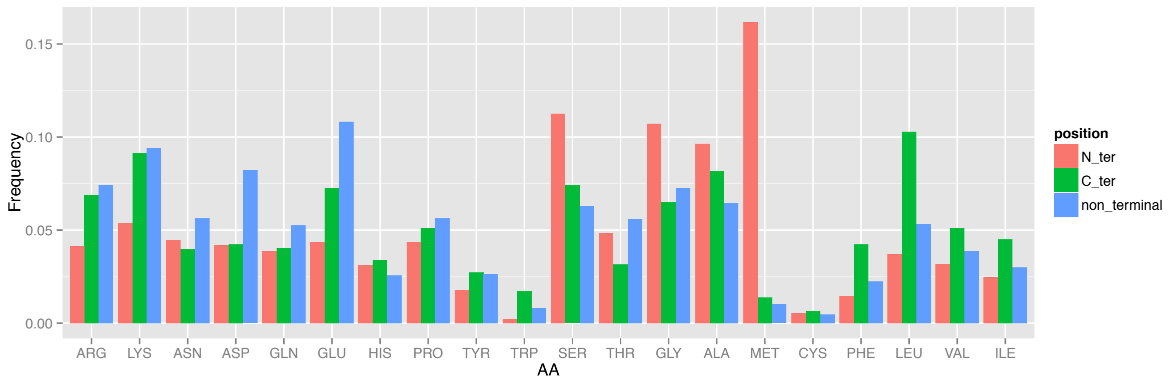

B

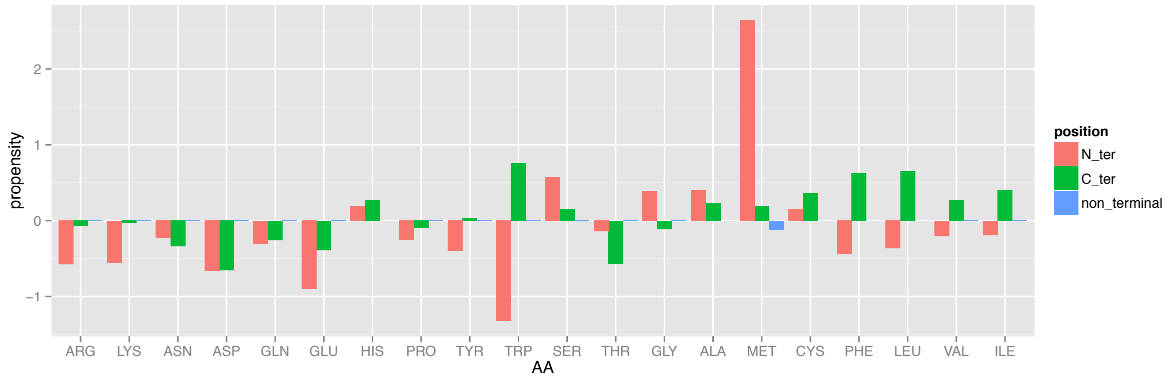

Figure D: Amino-acid composition of terminal and non-terminal residues. The analysis was restricted to the exposed residues of proteins from the MONOMER25 data set that have two genuine terminal residues (1891 proteins). A: raw percentages, B: propensities compared to the global composition of proteins

nterface ype	Complex type	Obligate class	Assembly pe	N-ter			C-ter		
				#res	F_Inter (%)	F_Rim (%)	#res	F_Inter (%)	F_Rim (%)
Biological	Homo	Obligate	dimer	2046	28	89	3322	32	80
Biological	Homo	Obligate	K-mer	1926	22	89	3075	26	80
Biological	Homo	Non-obligate	dimer	557	27	92	770	33	77
Biological	Homo	Non-obligate	K-mer	638	22	81	790	21	82
Biological	Hetero	Obligate	dimer	206	29	78	248	24	81
Biological	Hetero	Obligate	K-mer	638	22	81	790	21	82
Biological	Hetero	Non-obligate	dimer	422	25	81	502	25	71
Biological	Hetero	Non-obligate	K-mer	679	21	86	821	27	84
Crystal	Homo			1111	12	87	1536	14	82
Crystal	Hetero			559	10	89	672	8	87

Table S1. Involvement at interfaces and rim region for terminal residues of the DIMER70 data set. #res: number of terminal residues analyzed, F_inter: fraction of terminal residues involved at interfaces, F_rim: fraction of terminal residues in the rim of the regions, among those involved at interfaces.

nterface type	Complex type	Obligate class	Assembly type	N-ter			C-ter		
				#res[1]	F_inter (%)[1]	F_rim (%)[2]	#res	F_inter (%)	F_rim (%)
Biological	Homo	Obligate	dimer	431	27	89	660	33	78
			K-mer	206	23	89	286	28	79
		Non-obligate	dimer	131	27	86	194	32	78
			N-mer	79	30	96	99	26	89
	Hetero	Obligate	dimer	53	24	62	71	23	88
			N-mer	63	35	91	89	30	81
		Non-obligate	dimer	116	28	85	145	34	78
			N-mer	71	31	73	89	38	88
Crystal	Homo			59	12	100	105	14	93
	Hetero			39	2.5	100	45	9	100

Table S2. Involvement at interfaces and rim region for terminal residues of the MONOMER25 data set. #res: number of terminal residues analyzed, F_inter: fraction of terminal residues involved at interfaces, F_rim: fraction of terminal residues in the rim of the regions, among those involved at interfaces.

BIOLOGICAL COMPLEXES

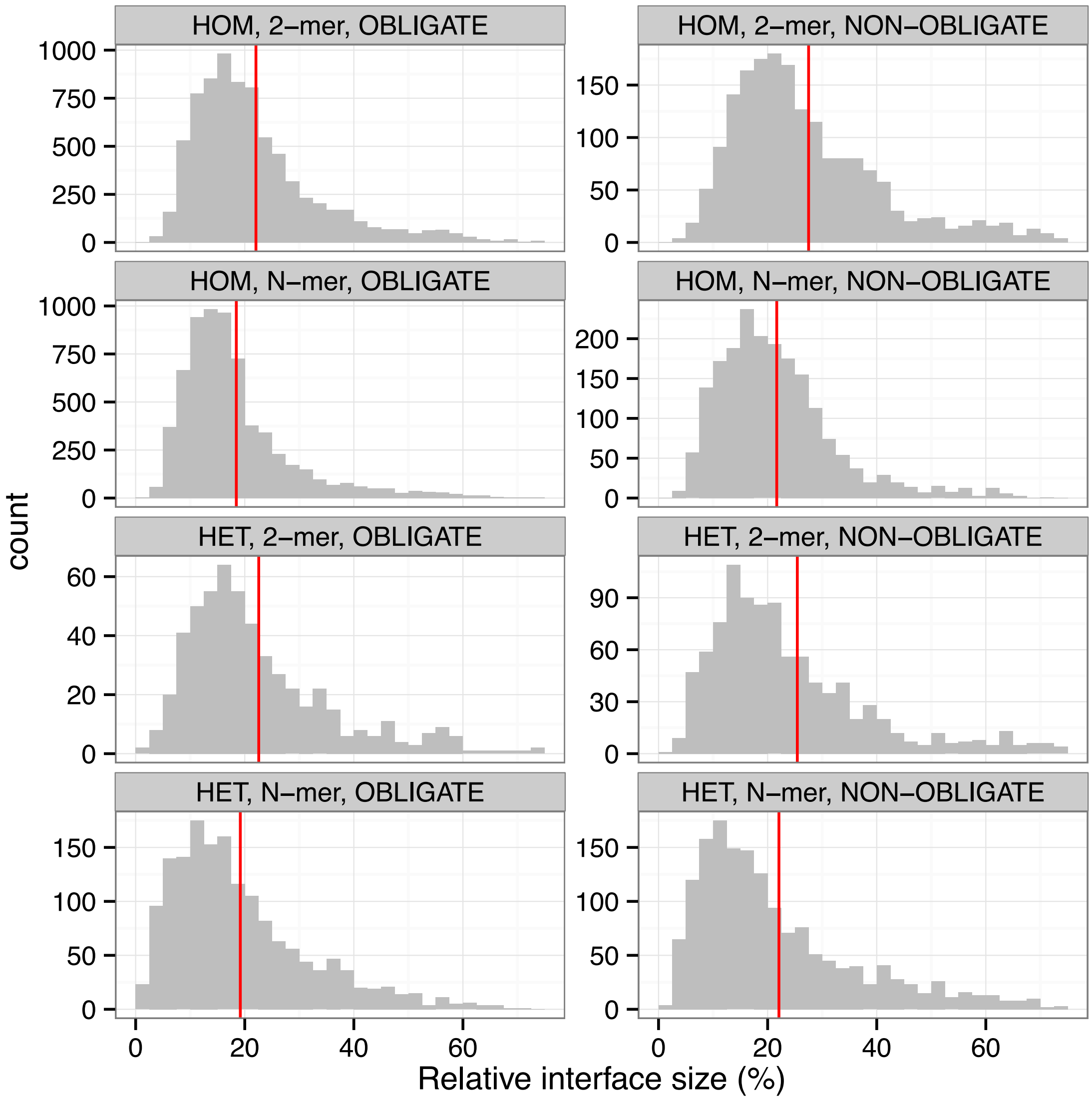

CRYSTAL CONTACTS

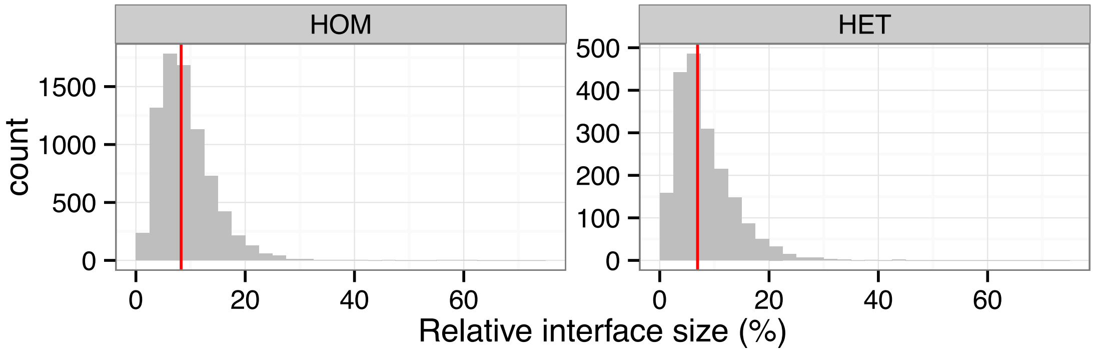

Figure E. Size of interfaces in complexes of different classes in the DIMER70 dataset. The size is expressed as the ratio between the number of residues at the interface and the total number of residues in each protein. Red vertical lines indicate the means of each distribution. HOM: homo-complexes, HET: hetero-complexes.

BIOLOGICAL COMPLEXES (DIMER70)

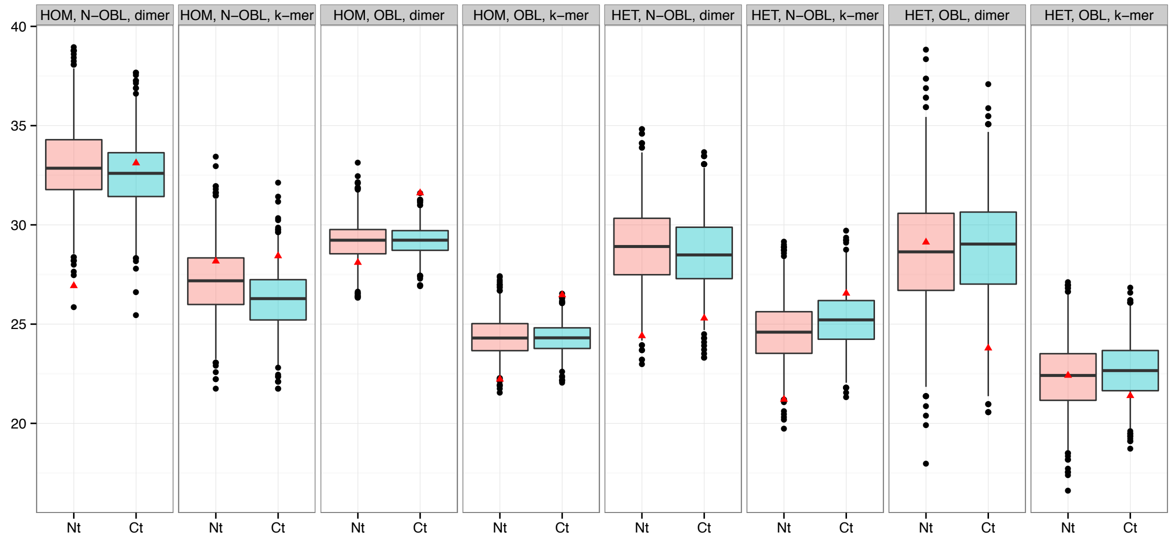

CRYSTAL CONTACTS (DIMER70)

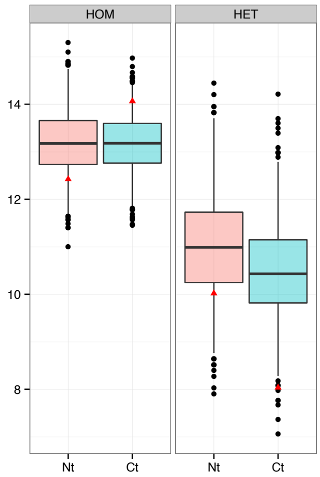

Figure F: Terminal residues are not over- or under-represented at protein-protein interfaces in the DIMER70 dataset. Each box plot displays the distribution of simulated values of F_inter (fraction of terminal residues at interfaces) computed from 1000 random data sets. The edges of the boxes correspond to the 25th and 95th percentiles, the notches extend from the 1st to the 99th, and outliers are black points. Observed fractions are depicted as red triangles.

BIOLOGICAL COMPLEXES (MONOMER25)

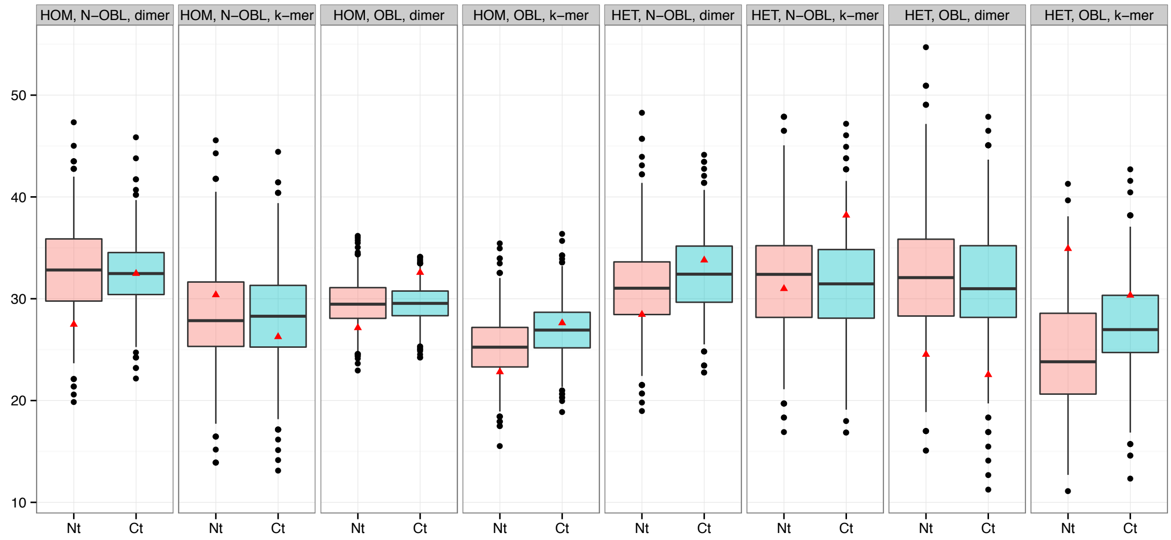

CRYSTAL CONTACTS (MONOMER25)

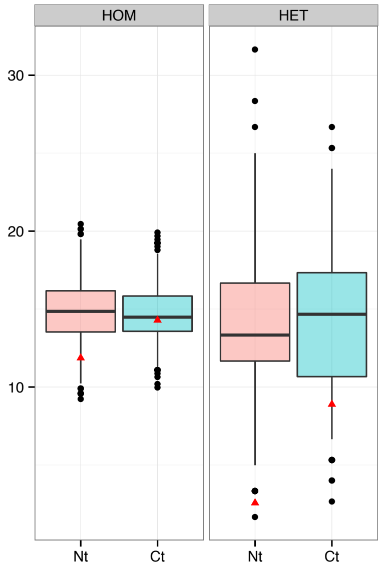

Figure G: Terminal residues are not over- or under-represented at protein-protein interfaces in the MONOMER25 dataset. Observed and simulated F_inter (fraction of terminal residues at interfaces).

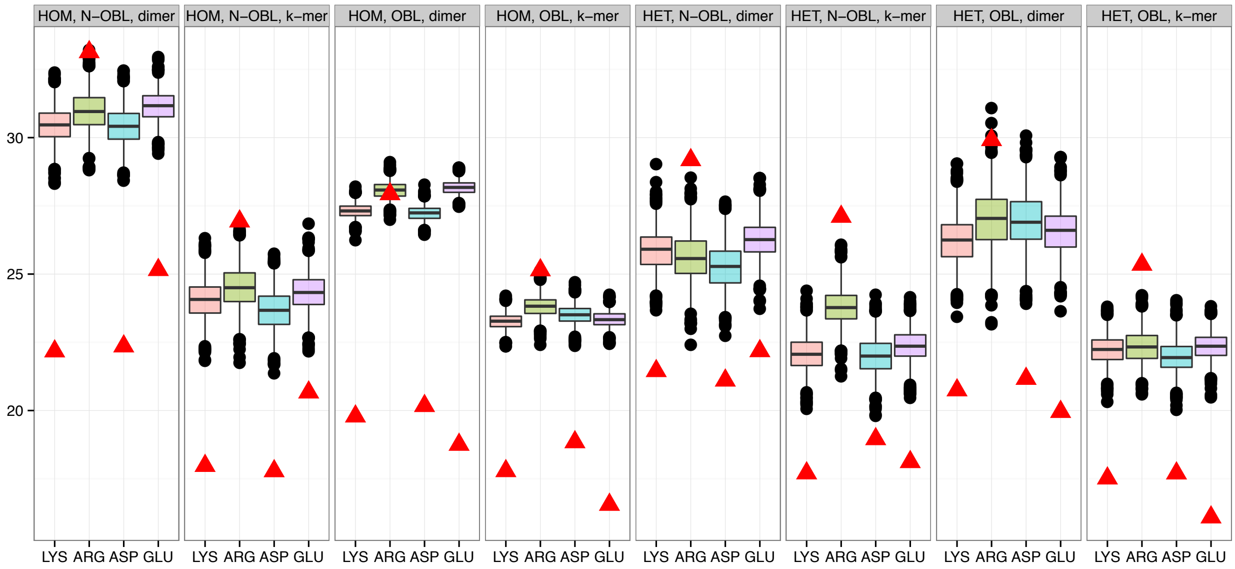

Figure H: Charged residues are under-represented (LYS, ASP, GLU) or over-represented (ARG) in interfaces in the MONOMER25 dataset. Observed and simulated F_inter (fraction of charged residues in interfaces) computed from 1000 random data sets. Pink boxes: lysine residues, green boxes: arginine residues, blue boxes: aspartate residues, purple boxes: glutamate residues.

BIOLOGICAL COMPLEXES (MONOMER25)

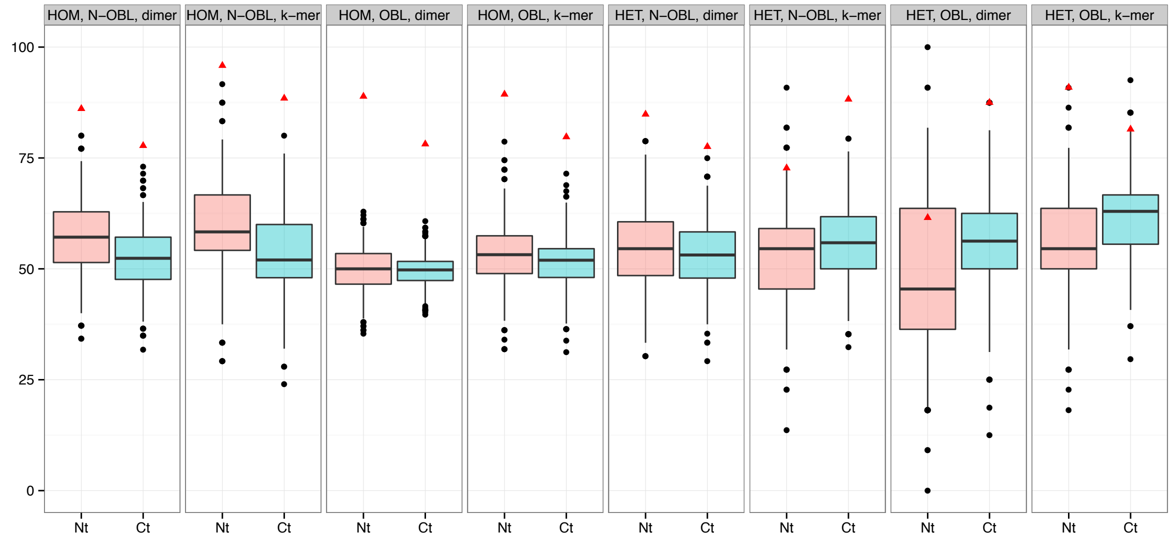

CRYSTAL CONTACTS (MONOMER25)

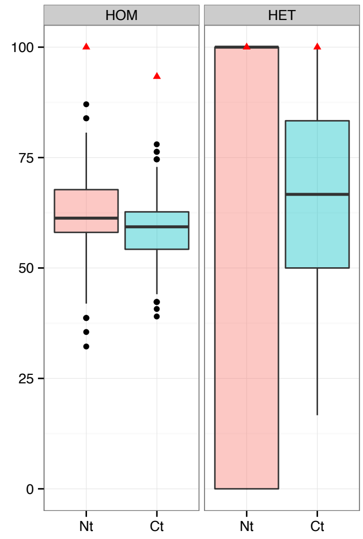

Figure I: Terminal residues are over-represented in the rim regions in the MONOMER25 dataset. Observed and simulated F_rim (fraction of terminal residues in rim regions, among those at the interface).

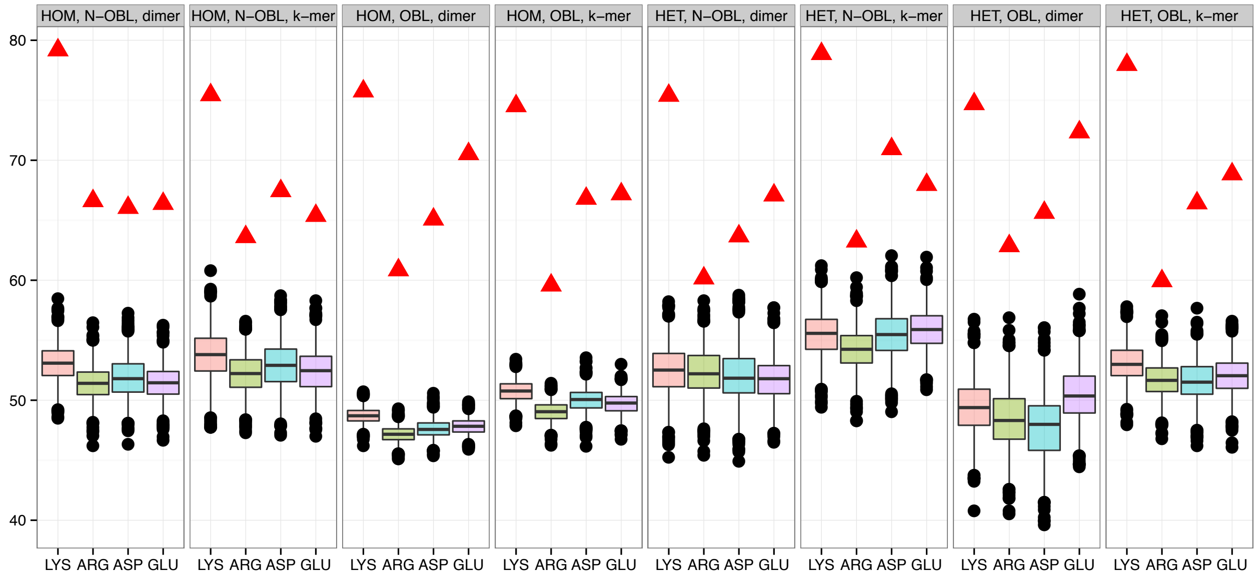

Figure J: Charged residues are over-represented in rim the MONOMER25 dataset. Observed and simulated F_rim (fraction of charged residues in the rim among those at the interfaces) computed from 1000 random data sets. Pink boxes: lysine residues, green boxes: arginine residues, blue boxes: aspartate residues, purple boxes: glutamate residues.

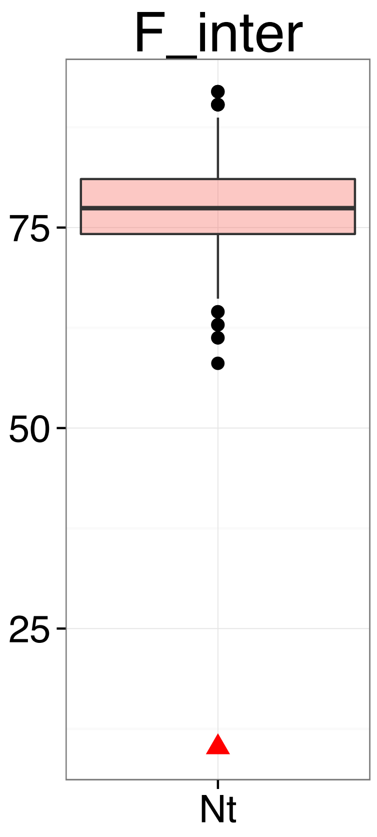

Figure K: N-terminal residues are underrepresented in protein-DNA interfaces, even after removing partial structures. Data are collected on a list of 49 proteins.
